# Malignancy Detection Rates of [^68^Ga]Ga-SSO120 PET/CT in Patients with Small Cell Lung Cancer and Large Cell Neuroendocrine Lung Cancer

**DOI:** 10.2967/jnumed.125.270233

**Published:** 2026-04

**Authors:** Tine N. Christensen, Seppo W. Langer, Danijela Dejanovic, Nicholas Gillings, Jacob Madsen, Gitte Persson, Sune H. Keller, Elisabeth Albrecht-Beste, Barbara M. Fischer

**Affiliations:** 1 Department of Clinical Physiology and Nuclear Medicine, Copenhagen University Hospital, Rigshospitalet, Copenhagen, Denmark;; 2 Department of Oncology, Copenhagen University Hospital, Rigshospitalet, Copenhagen, Denmark;; 3 Department of Clinical Medicine, University of Copenhagen, Copenhagen, Denmark; and; 4 Department of Oncology, Copenhagen University Hospital, Herlev Hospital, Herlev, Denmark

**Keywords:** [^68^Ga]Ga-SSO120 PET/CT, LCNEC, [^177^Lu]Lu-SSO110 radiopharmaceutical therapy, SCLC, SSTR2

## Abstract

The primary aim of this study was to investigate the malignancy detection rate of [^68^Ga]Ga-SSO120 ([^68^Ga]Ga-satoreotide trizoxetan) PET/CT in patients with small cell lung cancer (SCLC) and large cell neuroendocrine lung cancer (LCNEC). Secondary aims included investigations of lesion-based detection rates and quantification. **Methods:** In this prospective, phase 2, cross-sectional imaging trial, 21 [^68^Ga]Ga-SSO120 PET/CTs were performed for 19 patients. Overall detection rates, lesion-based detection rates, and number of identified lesions were compared between [^68^Ga]Ga-SSO120 PET and CT. The [^68^Ga]Ga-SSO120 SUV_max_, SUV_peak_, SUV_mean_, and tumor-to-liver ratios in malignant lesions and selected normal tissue were quantified. **Results:** Nineteen patients (16 with SCLC, 2 with LCNEC, and 1 with combined SCLC/LCNEC) were scanned during palliation chemotherapy (*n* = 11) or surveillance (*n* = 8). [^68^Ga]Ga-SSO120–detectable lesions were identified in 18 patients (95%). Sensitivity in the lungs, regional lymph nodes, and extrathoracic regions was 82%, 83%, and 93%, respectively. [^68^Ga]Ga-SSO120 PET detected significantly fewer lesions than did CT (*P* = 0.037), particularly small lung lesions, regional lymph nodes, and liver lesions. In contrast, [^68^Ga]Ga-SSO120 PET identified 10 additional metastases in 5 patients (bone, 7; cerebellum, 1; subcutaneous, 2). SUV_max_ (median, 7.4; range, 4.6–26.4) was not significantly associated with time since diagnosis, time since last chemotherapy, number of treatment cycles, or current progression. **Conclusion:** [^68^Ga]Ga-SSO120 PET/CT successfully visualized SCLC and LCNEC lesions during and after chemotherapy. Therapeutic studies with [^177^Lu]Lu-SSO110 ([^177^Lu]Lu-satoreotide tetraxetan), the theranostic companion of [^68^Ga]Ga-SSO120, PET are warranted.

## Visual Abstract

**Figure:**
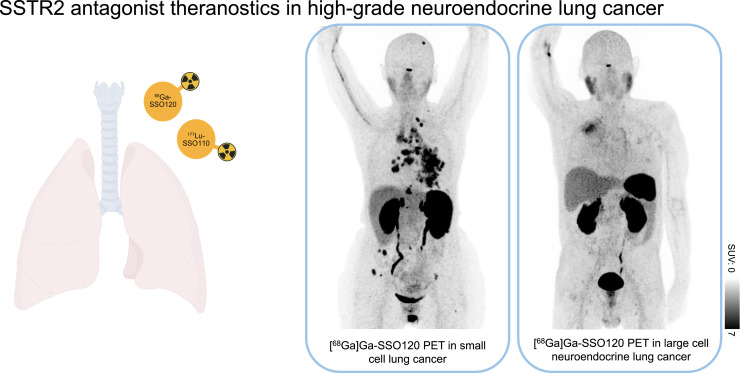

Small cell lung cancer (SCLC) is an aggressive cancer, accounting for approximately 12% of lung cancer cases worldwide (1). Treatment options are limited, and the prognosis is poor (2–4). Large cell neuroendocrine lung cancer (LCNEC) shares similarities with SCLC and non–small cell lung cancer (NSCLC), and both LCNEC and SCLC are characterized as high-grade neuroendocrine carcinomas of the lungs (5). LCNEC is rare and accounts for approximately 0.6% of lung cancers (1). Evidence for optimal treatment regimens is sparse, and the recommendations for treatment follow those for SCLC or NSCLC, in accordance with the individual molecular testing and genomic features (4,6).

Like other neuroendocrine neoplasms, a substantial number of SCLCs and LCNECs express somatostatin receptor 2 (SSTR2) (2,5,7). Molecular imaging and radiopharmaceutical therapy (RPT) with SSTR2 agonists (e.g., DOTATOC, DOTANOC, DOTATATE) for neuroendocrine tumors (NETs) is well established (6,8), and investigation of SSTR2 imaging and RPT in SCLC and LCNEC seems obvious. However, results show considerable interpatient variation, with limited positivity for SSTR imaging and poor responses to RPT (9).

The development of SSTR2 antagonists may potentially overcome this challenge. Studies have shown that SSTR2 antagonist imaging has a higher tumor-to-background ratio and sensitivity compared with SSTR2 agonist imaging (10–12). SSTR2 antagonists bind to both active and inactive receptors and stay on the receptor longer, giving hope that imaging and RPT may benefit patients with cancers with lower SSTR2 expression than NETs, such as SCLC and LCNEC.

[^68^Ga]Ga-SSO120 and [^177^Lu]Lu-SSO110 (also known as [^68^Ga]Ga-satoreotide trizoxetan/[^177^Lu]Lu-satoreotide tetraxetan, [^68^Ga]Ga-OPS202/[^177^Lu]Lu-OPS201, and [^68^Ga]Ga-NODAGA-JR11/[^177^Lu]Lu-DOTA-JR11) are a theranostic pair of SSTR2 antagonists. They share the same SSTR2 antagonist (satoreotide, JR11), but the conjugated chelator and the radiometal differ. [^68^Ga]Ga-SSO120 has shown an acceptable safety profile, and the effective dose (0.024 mSv/MBq) is comparable to that of [^68^Ga]Ga-labeled SSTR2 agonists with the highest mean dose coefficients in the bladder, kidneys, and spleen (13). It was recently demonstrated that the sensitivity of [^68^Ga]Ga-SSO120 PET/CT is comparable to that of [^18^F]FDG PET/CT for the initial staging of SCLC (14).

The aim of this study was to investigate the uptake of [^68^Ga]Ga-SSO120 in patients with high-grade neuroendocrine lung cancer, as a step toward determining optimal timing of [^177^Lu]Lu-SSO110 RPT. Secondary aims included lesion-based evaluations compared with standard imaging and quantification of malignant lesions and background tissue. To our knowledge, this is the first prospective study that investigates [^68^Ga]Ga-SSO120 PET/CT in patients with either SCLC or LCNEC.

## MATERIALS AND METHODS

### Patients

In this prospective clinical cross-sectional imaging study, patients with histologically confirmed SCLC, LCNEC, or combined SCLC/LCNEC were included at any treatment phase. Patients were excluded if the site of the primary tumor was unknown or if they had prior somatostatin analog treatment, prior radiotherapy to lung targets, complete response to therapy, or other active malignancies.

Patients were recruited from 2 university hospitals in Denmark—Rigshospitalet and Herlev Hospital—between September 2023 and April 2024. Approval was granted by the Danish Medical Research Ethics Committees and the Danish Medicines Agency (date 10/05/2023/EUCT number 2023-503362-24-01) and institutional review boards. The study was performed in accordance with the principles of the Declaration of Helsinki and monitored by the Danish GCP-unit at Copenhagen University Hospital. The trial was registered in the Clinical Trials Information System on May 10, 2023 (EUCT 2023-503362-24-01).

All patients provided written informed consent for participation and for publication of images from the scans. Health data, including baseline data, pathology, imaging reports, and previous and current treatments were collected from the medical records. Tumor staging was based on the *TNM Classification of Malignant Tumours, 8th edition.*

### Imaging

All patients underwent a [^68^Ga]Ga-SSO120 PET/CT scan at Rigshospitalet. [^68^Ga]Ga-SSO120 was prepared for administration at the Department of Clinical Physiology and Nuclear Medicine, Cyclotron and Radiochemistry Section, Rigshospitalet, in accordance with the directions in the investigational medicinal product dossier.

Before the PET/CT images were obtained, 150 ± 50 MBq of [^68^Ga]Ga-SSO120 was injected as a single dose through a venous catheter. After 60 ±10 min of rest, a static whole-body PET/CT was performed on a Biograph Vision 600 Edge PET/CT or a Biograph Vision 600 PET/CT scanner (Siemens Healthineers) in combination with a diagnostic, contrast-enhanced CT. Both scanners were rigorously cross-calibrated using a previously published procedure (15).

Patient weight was measured in the department, the injected activity was corrected for residual activity in the syringe, and time of injection and start time of the scan were recorded digitally. PET images were reconstructed using ordered-subset expectation maximization with point-spread function modeling (4 iterations, 5 subsets) and time-of-flight reconstruction.

Patients were interviewed before leaving the department and 24 h after injection of [^68^Ga]Ga-SSO120 to assess for any adverse events.

### Image Analysis

The PET/CT scans were evaluated on a Mirada Medical Ltd. XD 3.6.9 workstation by 2 board-certified nuclear medicine physicians with more than 10 y of experience each. The CT scans were evaluated for project purposes by a board-certified radiologist. Up to 5 lesions in the lungs, 3 in regional lymph nodes, 3 in the liver, 3 in the bones, and 5 in other anatomic sites were evaluated. Lesions were scored as being malignant, equivocal, or benign. Disagreement between the observers related to TNM classification was resolved by consensus.

[^68^Ga]Ga-SSO120 uptake was quantified in malignant/equivocal lesions and in background organs. Malignant/equivocal lesions were segmented using an SUV greater than 4.0 as the threshold, as suggested for other tracers (16,17). The SUV_max_ and average SUV (SUV_mean_) were collected within the region of interest (ROI). SUV_peak_ (average of SUV in a sphere with a volume of 1 cm^3^, placed in the ROI to maximize the value) was collected only in ROIs larger than 1 cm^3^. Maximum tumor-to-liver ratios were calculated as the SUV_max_ normalized to SUV_mean_ in the liver, measured in a sphere of 3 cm^3^ in the right liver lobe (14). Because of the high physiologic uptake of [^68^Ga]Ga-SSO120 in the adrenal glands, adrenal metastases were excluded in the quantitative analysis.

In selected normal tissue, spheric ROIs were manually placed in the area with the highest uptake. An ROI of 3 cm in diameter was preferred; in smaller organs or tissues, an ROI of 1 or 2 cm in diameter was used. The pituitary glands were segmented with a fixed SUV threshold of greater than 2.5. For lung measurements, the ROI was placed in a lower lobe, whereas for liver measurements, it was positioned in the right lobe. Uptake in bone marrow was measured in vertebra L4 (preferably); if not feasible, an adjacent vertebra was used. Uptake in bone was measured in the femoral bone. Known malignant and benign changes were avoided, as were the kidney pelvises. In these ROIs, SUV_max_ and SUV_mean_ were measured. SUV_peak_ was measured only in ROIs larger than 1 cm^3^. In the intestines, only SUV_max_ was considered reliable.

### Endpoints and Reference Standard

The primary endpoint was the fraction of patients with at least 1 lesion detectable by [^68^Ga]Ga-SSO120 PET. Secondary endpoints included comparisons of the number and sites of [^68^Ga]Ga-SSO120 PET–identified malignant lesions with lesions identified by standard imaging, as well as quantification of malignant lesions and normal tissue.

Standard imaging, including CT and [^18^F]FDG PET/CT, performed within 14 d of the [^68^Ga]Ga-SSO120 PET/CT was considered the reference standard.

### Statistical Analysis

Statistical analysis was performed using SPSS Statistics 29.0.1.0 (IBM) and R version 4.3.0 (R Foundation).

The study size was chosen with the theoretic expectation that 50% of patients would exhibit [^68^Ga]Ga-SSO120–avid uptake. The inclusion of 20 patients with evaluable [^68^Ga]Ga-SSO120 PET/CT scans would correspond to a sensitivity of 50% with a 95% CI of 27%–73%, which was considered acceptable.

Patient characteristics, tumor characteristics, patient-based detection rate, and qualitative parameters of [^68^Ga]Ga-SSO120 PET were analyzed descriptively. The number of identified malignant lesions from [^68^Ga]Ga-SSO120 PET/CT was compared with results from standard imaging using Wilcoxon signed rank test, and lesion-based sensitivity and specificity were calculated in 5 anatomic regions (lung, regional lymph nodes, extrathoracic metastases, liver, and bone). Associations between the highest patient-based [^68^Ga]Ga-SSO120 SUV_max_ and time since diagnosis, time since therapy, number of chemotherapy cycles, and current CT response was analyzed using Kendall τ-correlation and Welch test. A *P* value of less than 0.05 was considered significant.

## RESULTS

Between September 2023 and April 2024, 21 patients were selected for study inclusion. Of these, 2 withdrew their consent before undergoing the scan because of the deterioration of their general condition. In total, 21 [^68^Ga]Ga-SSO120 PET/CTs were performed in 19 patients. Two patients were scanned twice; the first [^68^Ga]Ga-SSO120 PET/CT was used for the analysis, and the second only for describing intrapatient changes.

The [^68^Ga]Ga-SSO120 PET/CTs were performed 58–69 min (median, 60 min) after the injection of 100–192 MBq of [^68^Ga]Ga-SSO120 (median, 162 MBq). All patients had diagnostic CT performed simultaneously, and no additional imaging was available within 14 d.

All patients with SCLC were initially staged as having extensive disease. The patients with LCNEC or combined disease had been diagnosed with stages IIb, IIIb, or IVa. The [^68^Ga]Ga-SSO120 PET/CT scans were performed 2–11 mo (median, 4.5 mo) after initial diagnosis, during first-line chemotherapy (*n* = 9), during reinduction chemotherapy (*n* = 2), or during surveillance (*n* = 8). The [^68^Ga]Ga-SSO120 PET/CTs were performed 1–247 d (median, 31 d) after their last treatment. None had received radiotherapy or immunotherapy.

At the time of the [^68^Ga]Ga-SSO120 PET/CT, 11 patients had progression on CT, whereas 8 patients showed a response, including 2 patients with SCLC who had a complete response of their extrathoracic metastases. Patient characteristics, including TNM stage, are presented in Table 1.

**TABLE 1.** Patient Characteristics (*n* = 19)

Characteristic	Value
Age (y)	70 (54–90)
Sex	
Male	11
Female	8
Histology	
SCLC	16
LCNEC	2
SCLC/LCNEC	1
TNM classification (restaging)	
IIIa	1
IIIb	1
IIIc	1
IVa	3
IVb	13
Chemotherapy	
Carboplatin plus etoposide	16
Etoposide	3
No. cycles of chemotherapy	
3	5
4	10
5	0
6	2
7	2

Data are expressed as median, followed by range in parentheses, or number.

### Patient-Based [^68^Ga]Ga-SSO120 PET Detection Rate

**FIGURE 1.**
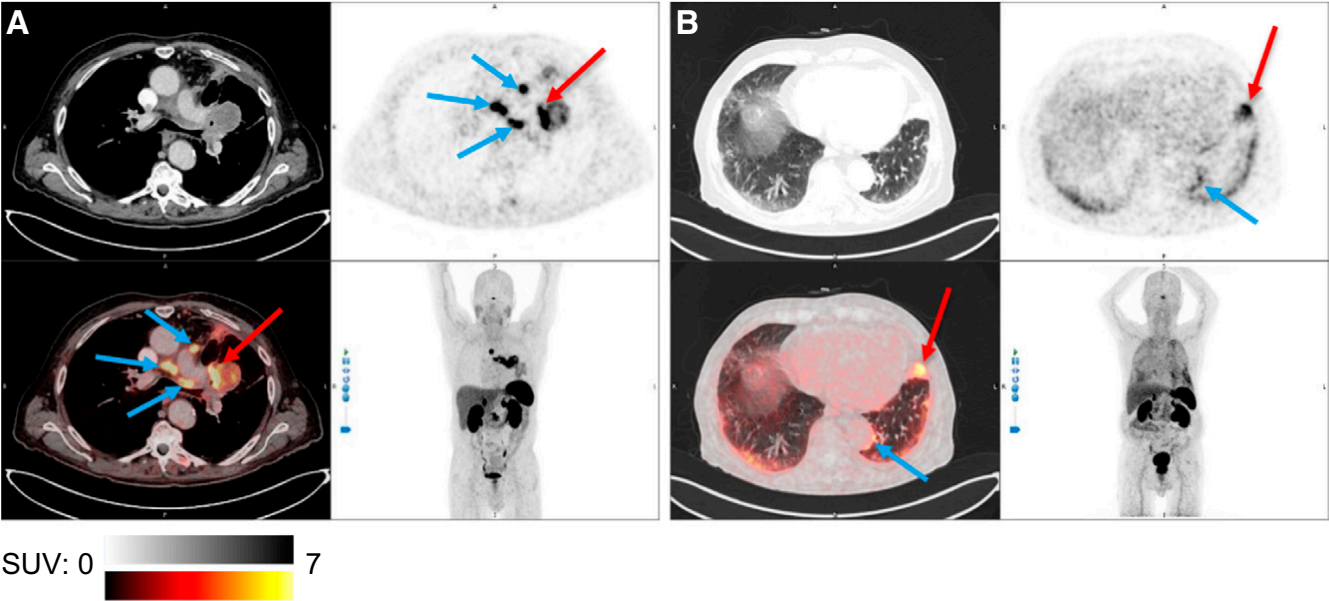
Examples of [^68^Ga]Ga-SSO120 PET/CT. Transversal CT (upper left), [^68^Ga]Ga-SSO120 PET (upper right), fused [^68^Ga]Ga-SSO120 PET/CT (lower left), and maximum-intensity-projection (lower right) images are shown. (A) High [^68^Ga]Ga-SSO120 uptake in primary SCLC tumor (red arrow; SUV_max_, 26.4) and mediastinal lymph nodes (blue arrows; SUV_max_, 19.4) 3 mo after end of 4 cycles of carboplatin plus etoposide. (B) High [^68^Ga]Ga-SSO120 uptake in primary LCNEC tumor (red arrow; SUV_max_, 8.1) and metastatic pleura (blue arrow; SUV_max_, 8.3) after third cycle of etoposide as monotherapy. (A and B) Physiologic uptake in pituitary gland, salivary glands, spleen, liver, adrenal glands, kidneys, and bladder is shown.

[^68^Ga]Ga-SSO120 PET identified at least 1 malignant lesion in 18 (95%) of 19 patients (Fig. 1). In 1 patient with SCLC, [^68^Ga]Ga-SSO120 PET detected no lesions, whereas CT revealed malignancy in the lung and mediastinum. Maximum-intensity-projection images for each patient are provided in Supplemental Figure 1 (available at http://jnm.snmjournals.org).

### Lesion-Based [^68^Ga]Ga-SSO120 PET Analyses

Significantly more malignant lesions were detected by CT than [^68^Ga]Ga-SSO120 PET (*P* = 0.037). Differences were mostly seen in the lymph nodes (*P* = 0.011) and liver (*P* = 0.020). Though some small lung lesions were missed by [^68^Ga]Ga-SSO120 PET, there were no significant differences in the number of detected lesions in the lungs (*P* = 0.559), bones (*P* = 0.496), or extrathoracic metastases (*P* = 0.093).

**FIGURE 2.**
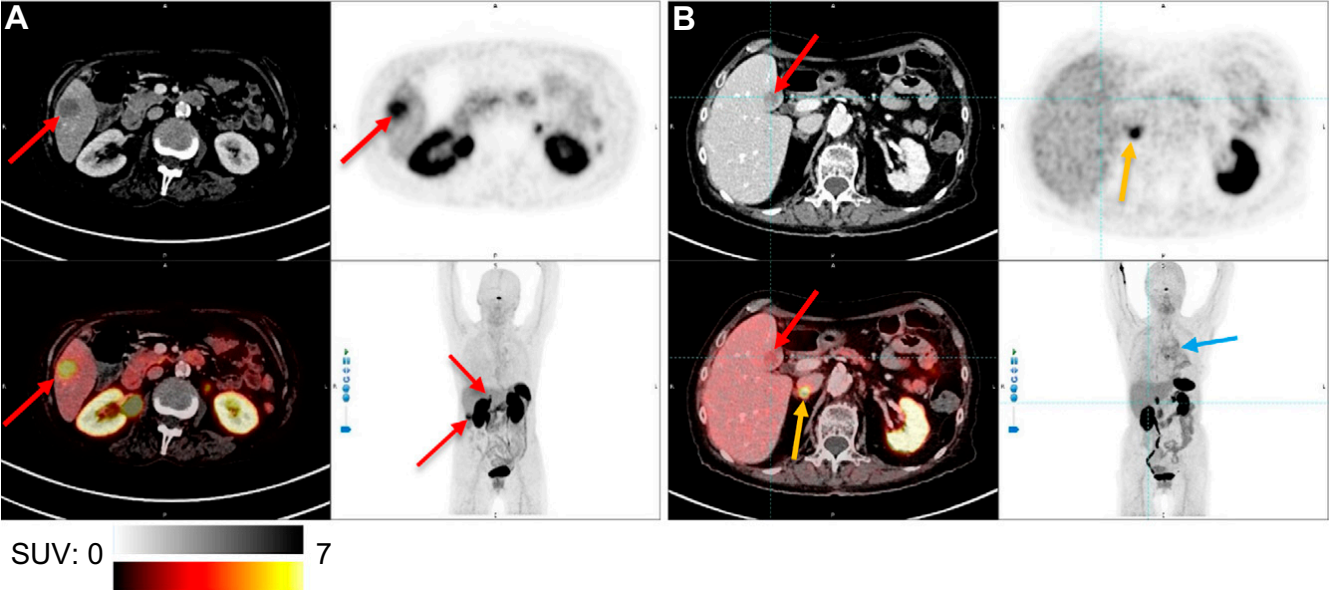
[^68^Ga]Ga-SSO120 PET/CT of liver metastases. Transversal CT (upper left), [^68^Ga]Ga-SSO120 PET (upper right), fused [^68^Ga]Ga-SSO120 PET/CT (lower left), and maximum-intensity-projection (lower right) images shown. (A) Two SCLC liver metastases (red arrows) were detected by [^68^Ga]Ga-SSO120 PET during reinduction therapy with carboplatin plus etoposide. (B) SCLC liver metastasis with no [^68^Ga]Ga-SSO120 uptake 3 mo after end of 4 cycles carboplatin plus etoposide. Primary tumor (blue arrow) was detectable (SUV_max_, 6.2). (A and B) Physiologic uptake in pituitary gland, spleen, liver, adrenal glands (yellow arrows), kidneys, and bladder is also shown.

In the liver, 6 patients had undetected metastases by [^68^Ga]Ga-SSO120 PET (Fig. 2). In the kidneys, metastasis was undetected by [^68^Ga]Ga-SSO120 PET in 1 patient. In contrast, [^68^Ga]Ga-SSO120 PET identified new metastases that had not been detected by CT in 5 patients (7 bone, 2 subcutaneous, and 1 cerebellar).

Lesion-based sensitivities in the lungs, regional lymph nodes, extrathoracic metastases, bone, and liver were 82% (14/17), 83% (10/12), 93% (13/14), 89% (8/9), and 25% (2/8), respectively. Specificities were 0% (0/1), 100% (5/5), 60% (3/5), 70% (7/10), and 100% (11/11), respectively. One lung lesion and regional lymph nodes were classified as equivocal by CT in 1 and 2 patients, respectively, and not included in the analysis. Confusion matrices for the anatomic regions are available in Supplemental Table 1.

### [^68^Ga]Ga-SSO120 PET Quantification

In the 18 patients with detectable [^68^Ga]Ga-SSO120 uptake, 92 lesions were segmented. SUV_max_ was 4.6–26.4 (median, 7.4), with higher than local background uptake and liver uptake in all patients (Table 2; Supplemental Table 2).

**TABLE 2.** [^68^Ga]Ga-SSO120 Uptake Values in the Hottest Malignant Lesion

Variable	*n*	Median	Range	IQR
SUV_max_	18	7.4	4.6–26.4	6.2–10.8
SUV_peak_	14	5.7	4.1–14.1	4.5–7.0
SUV_mean_	18	4.9	4.2–8.5	4.6–5.2
TLR_max_	18	2.5	1.6–8.8	2.1–3.4
TLR_peak_	14	2.0	1.1–4.7	1.5–2.8
Mean SUV_mean_	18	4.6	4.2–6.4	4.4–4.9

IQR = interquartile range; TLR_max_ = maximum tumor-to-liver ratio; TLR_peak_ = peak tumor-to-liver ratio; Mean SUV_mean_ = average of SUV_mean_ in all lesions in each patient.

An SUV_max_ of greater than 10 was observed in 5 patients with SCLC: 1 patient had high [^68^Ga]Ga-SSO120 uptake in all lesions (lung, regional lymph nodes, subcutaneous, and bone metastases). Two patients had an SUV_max_ of greater than 10 in lung lesions and regional lymph nodes but a lower SUV_max_ (4–6) in bone metastases. Two patients had an SUV_max_ of greater than 10 only in either regional lymph nodes or bone metastases but a lower SUV_max_ (2.2–8.8) in lung lesions, lymph nodes, and bone metastases.

Of the visually detected lesions, 21 lesions in 10 patients had an SUV_max_ of less than 4 and were therefore not segmented. This included 7 lung lesions, 5 lymph nodes, 6 bone metastases, and 3 brain metastases. Although 13 of these lesions had [^68^Ga]Ga-SSO120 uptake above uptake in the liver, 8 lesions exhibited [^68^Ga]Ga-SSO120 uptake below uptake in the liver, including 4 small lung lesions, 3 brain metastases, and 1 bone metastasis.

### LCNEC

[^68^Ga]Ga-SSO120 PET detected malignancy in both patients with LCNEC and the 1 patient with combined LCNEC/SCLC, with an SUV_max_ in the lungs of 5.3, 7.5, and 8.3, respectively.

Immunohistochemical SSTR2 expression was analyzed in 1 patient. SSTR2 expression in the diagnostic cytologic sample from a hilar lymph node was negative; however, ipsilateral hilar lymph nodes were detectable (SUV_max_, 5.6).

### [^68^Ga]Ga-SSO120 PET Changes over Time

**FIGURE 3.**
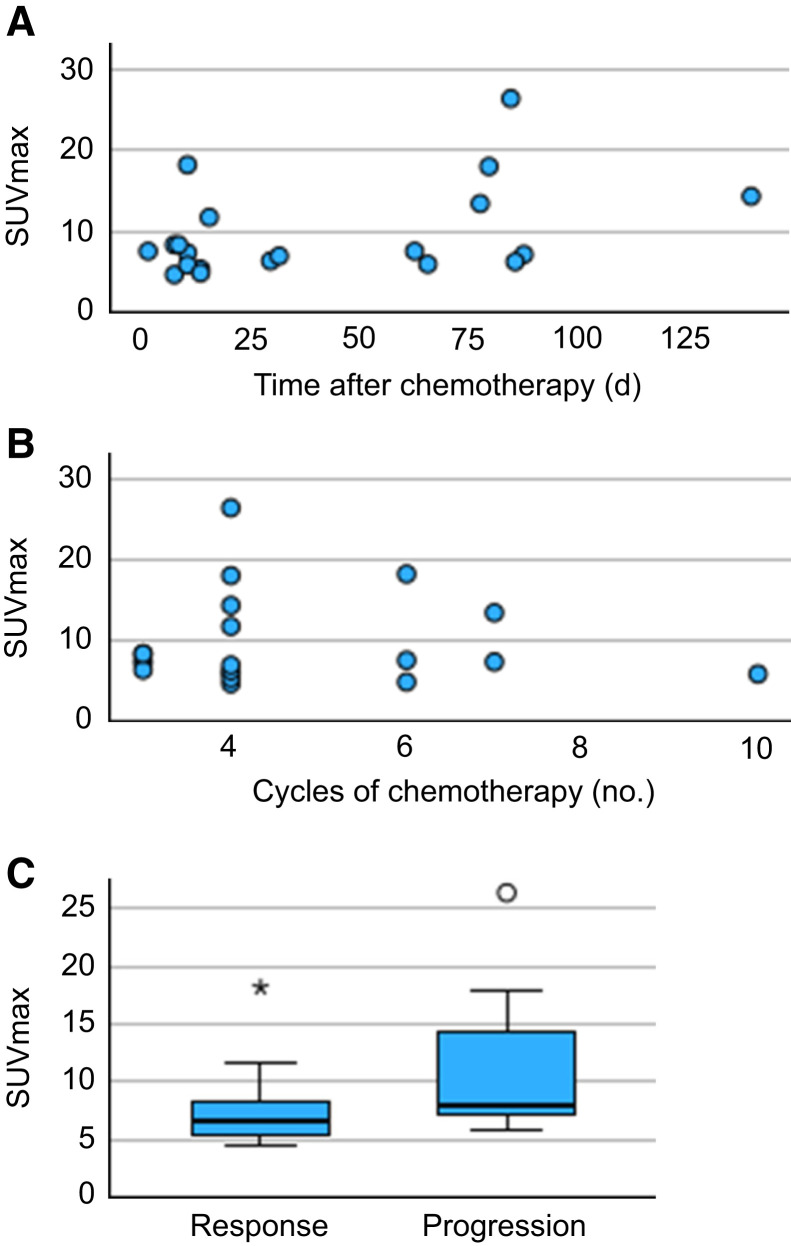
Associations between SUV_max_ and time since last chemotherapy (A), number of treatment cycles of chemotherapy that patient received before [^68^Ga]Ga-SSO120 PET/CT (B), and current CT evaluation (C).

SUV_max_ did not significantly correlate with time from diagnosis (Kendall τ = 0.095; 95% CI, −0.224 to 0.396; *P* = 0.559), interval from last treatment (Kendall τ = 0.209; 95% CI, −0.111 to 0.490; *P* = 0.204), or number of treatment cycles with chemotherapy (Kendall τ = −0.43; 95% CI, −0.351 to 0.274; *P* = 0.809). There was no significant difference in median SUV_max_ among patients whose tumors showed a response compared with those whose disease exhibited progression (6.6 vs. 7.9; *P* = 0.070) (Fig. 3).

**FIGURE 4.**
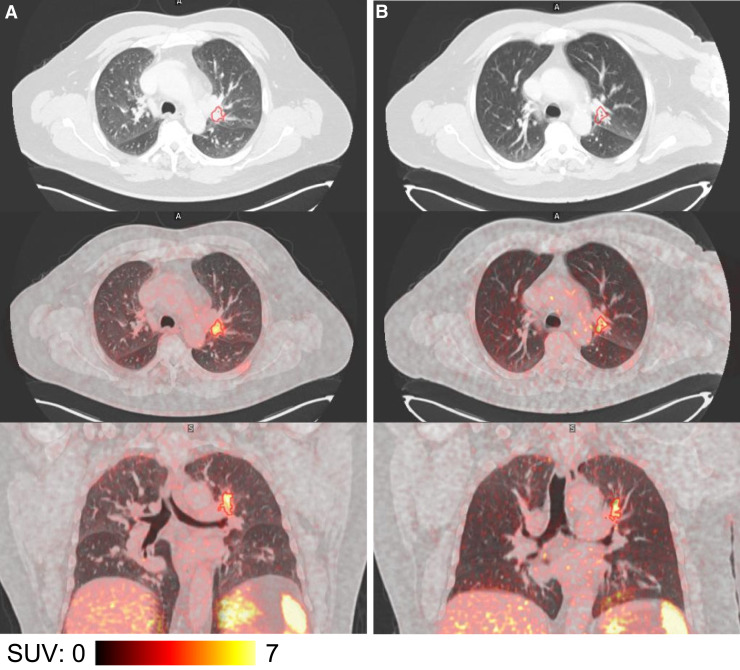
[^68^Ga]Ga-SSO120 PET/CT in patient with SCLC patient who was scanned twice 1.5 mo between and after 4 (A) and 6 cycles (B) of carboplatin plus etoposide. Panels shows transversal CT (upper row), fused transversal [^68^Ga]Ga-SSO120 PET/CT (middle row), and fused coronal [^68^Ga]Ga-SSO120 PET/CT images (lower row). Primary tumor is segmented by SUV of greater than 4 (red line). On CT, size of primary tumor was reduced from 23 × 16 mm to 14 × 11 mm. [^68^Ga]Ga-SSO120 PET showed no clear difference in visual appearance or PET quantification of primary tumor (SUV_max_, 8.8 vs. 9.6; SUV_mean_, 4.9 vs. 5.1).

Two patients with SCLC underwent [^68^Ga]Ga-SSO120 scans twice, with intervals of 1.5 and 6 mo between scans, respectively. In the first patient, the initial [^68^Ga]Ga-SSO120 PET/CT was performed after receiving 4 cycles of carboplatin and etoposide. Following a favorable response, the patient received additional 2 cycles of carboplatin and etoposide, after which the second scan was performed. In the second patient, the first scan was acquired during surveillance, 10 mo after completion of first-line chemotherapy. Because of disease progression, the patient was treated with reinduction chemotherapy. The second scan was performed after 6 additional cycles of carboplatin and etoposide. Despite additional chemotherapy and CT-confirmed tumor regression, [^68^Ga]Ga-SSO120 uptake remained unchanged (Fig. 4).

### Biodistribution of [^68^Ga]Ga-SSO120 Uptake

**FIGURE 5.**
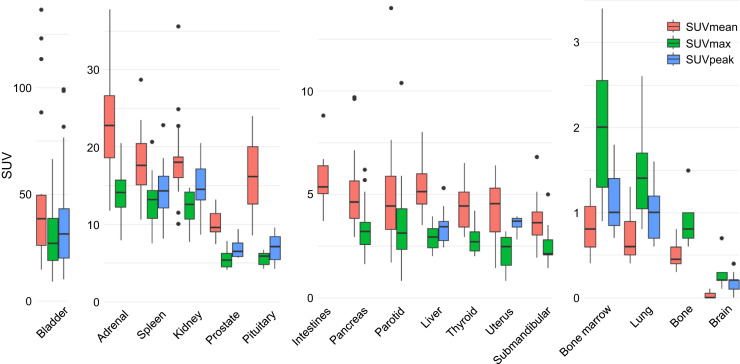
Box plot of SUVs in normal tissue. SUV_peak_ reported only for tissues with ROIs larger than 1 cm^3^.

[^68^Ga]Ga-SSO120 uptake was highest in the bladder, adrenal glands, spleen, kidneys, and pituitary gland. The remaining glands exhibited intermediate uptake. [^68^Ga]Ga-SSO120 uptake was lowest in the brain, lungs, bone, and bone marrow (Fig. 5; Supplemental Table 3). Notably, large interpatient variability was observed in the parotid glands.

### Adverse Events

Adverse events were reported in 8 patients during or within 24 h after the injection of [^68^Ga]Ga-SSO120. A temporary burning sensation during the injection was reported by 4 patients and categorized as a grade 1 adverse reaction. Eye flickering, nausea, fatigue, chest pain, and diarrhea (grades 1–2) were reported by 1 patient each. Because of the presence of other more likely causes or their late onset, these events were assessed as unlikely related or not related to [^68^Ga]Ga-SSO120 (Supplemental Table 4).

## DISCUSSION

In this prospective study, we reported several notable findings of [^68^Ga]Ga-SSO120 PET visualization in patients with SCLC and LCNEC. [^68^Ga]Ga-SSO120 PET visualized lesions in 15 of 16 patients with SCLC and in all 3 patients with LCNEC during and after chemotherapy. In all patients, [^68^Ga]Ga-SSO120 uptake was higher than background liver uptake, and 5 patients had a [^68^Ga]Ga-SSO120 SUV_max_ exceeding 10. To our knowledge, these are the first reported data on [^68^Ga]Ga-SSO120 PET/CT in patients with LCNEC and the first data on [^68^Ga]Ga-SSO120 PET/CT in patients with SCLC both during and after treatment.

Recently, a retrospective study showed a similarly high detection rate of baseline [^68^Ga]Ga-SSO120 PET/CT in patients with SCLC (14). In the previous study, 13 of 31 patients had high [^68^Ga]Ga-SSO120 uptake (mean SUV_max_, >10), a slightly higher percentage than in our study (SUV_max_, >10 in 5/16 patients). Although receptor expression is typically higher in well-differentiated tumors, this may not be the case for SSTR2 in SCLC, and high expression of SSTR2, measured by immunohistochemistry as well as [^68^Ga]Ga-SSO120 PET uptake, has been linked to poorer outcomes in patients with SCLC receiving standard therapy (18). The lower percentage of patients with high [^68^Ga]Ga-SSO120 uptake in the current study may reflect a selection bias, as those with early mortality, and perhaps the highest SSTR2 expression, were not included in the study.

Lesion-based detection rates evaluated by [^68^Ga]Ga-SSO120 PET compared with CT showed differences, particularly for liver metastases and smaller lesions. Histologic confirmation was neither feasible nor ethical, and CT, being the recommended imaging modality for treatment evaluation and surveillance in SCLC (3), was used as the reference. However, CT is not infallible, and the use of CT as the reference for malignancy has limitations. The discrepancies may therefore represent false negatives or false positives from either modality. Interestingly, [^68^Ga]Ga-SSO120 PET/CT slightly outperformed [^18^F]FDG PET/CT in detecting liver metastases in SCLC in a previous study (14); however, treatment effects may challenge the interpretation of both CT and [^68^Ga]Ga-SSO120 PET/CT. The clinical relevance of 10 additional metastases in 5 patients identified by [^68^Ga]Ga-SSO120 PET, but not seen on CT, is currently unknown.

Other limitations of our study include the small number of patients, particularly those with LCNEC. The patients in this cohort comprised a heterogeneous cohort and may not be representative of all patients with SCLC or LCNEC. All patients had undergone at least 3 cycles of chemotherapy. Therefore, it is possible that patients with the most aggressive cancers and those who did not respond well to first-line therapy were underrepresented. Additionally, none of the patients had received second-line therapy, radiotherapy, or immune checkpoint inhibitors, the latter of which was not approved for the treatment of SCLC by the Danish Medical Council at the time. The lesion count evaluation was limited by a fixed maximum in each region, although exceeding these thresholds would not have clinical relevance. No baseline [^68^Ga]Ga-SSO120 PET/CT was conducted; thus, the individual treatment effect of [^68^Ga]Ga-SSO120 uptake remains unknown.

[^68^Ga]Ga-SSO120 is safe to use with no grade 3 or 4 adverse events reported in the current study nor previously (13,19,20). A burning sensation during injection of [^68^Ga]Ga-SSO120 was described in 4 of 19 patients. In the current study, the [^68^Ga]Ga-SSO120 SUV_max_ in normal tissue was higher in all organs, except the lungs, compared with the SUV_max_ reported in a previous [^68^Ga]Ga-SSO120 PET/CT study involving patients with NETs (13). Differences in disease severity, treatment history (chemotherapy vs. prior somatostatin analog therapy), tracer peptide levels (13), and reconstruction methods, as well as technical differences of the PET scanners, may have influenced quantification significantly. Further, the ROI placement strategy varied, as the ROI was placed manually in areas with highest uptake in the current study, and the previous study used volumes near lesions.

Despite some differences in the detection rates of [^68^Ga]Ga-SSO120 PET and CT, particularly concerning liver metastases and smaller lesions, we demonstrated that [^68^Ga]Ga-SSO120 binds sufficiently to SCLC and LCNEC to enable visualization with PET, even after chemotherapy. Being part of a theranostic pair, [^68^Ga]Ga-SSO120 PET is expected to predict response to RPT with the companion treatment [^177^Lu]Lu-SSO110 (12). The exploration of new and potentially more effective treatment strategies is highly relevant, particularly for patients with SCLC and LCNEC with limited treatment options.

In the current study, SUV_max_ was not correlated with the number of chemotherapy cycles or time from chemotherapy. RPT with [^177^Lu]Lu-SSO110 could be investigated after first-line chemotherapy or at a later point, such as at the time of relapse. [^177^Lu]Lu-SSO110 has shown an acceptable safety profile in a phase 1/2 study of patients with NETs, although grade 3 and 4 hematologic toxicities were reported (21). The highest organ-absorbed doses of [^177^Lu]Lu-SSO110 were reported in the kidneys, liver, spleen, and bone marrow (22). In the current study, high uptake of [^68^Ga]Ga-SSO120 was also found in the pituitary and adrenal glands, suggesting that the biodistribution of [^177^Lu]Lu-SSO110 in these glands should be considered in future studies.

Optimal patient selection for [^177^Lu]Lu-SSO110 RPT remains to be defined. Uptake above normal liver uptake (Krenning score ≥ 3) has usually guided eligibility of patients with NETs for RPT with SSTR2 analogs (23). In the current study, all patients with detectable tumors had at least 1 lesion with uptake above liver uptake. However, because of different biodistribution and kinetics, results from other SSTR2 PET and RPT studies may not apply to [^68^Ga]Ga-SSO120 PET or [^177^Lu]Lu-SSO110 RPT. In patients with NETs, uptake of [^177^Lu]Lu-SSO110 in tumors was 5.4 times higher than [^68^Ga]Ga-SSO110 uptake, with highest ratio (8.0×) in lesions with lower [^68^Ga]Ga-SSO110 uptake (SUV_peak_, <10) (24). It has therefore been suggested that low [^68^Ga]Ga-SSO110 uptake should not exclude patients from [^177^Lu]Lu-SSO110 RPT. The SSTR Reporting and Data System incorporates clinical lesion assessment, potentially improving patient selection (23).

Currently, results from a phase 1 trial that investigated [^177^Lu]Lu-SSO110 RPT as maintenance therapy in addition to immune checkpoint inhibitors in patients with extensive stage SCLC (ACTRN12623000185662) are pending. Further, phase 1b studies investigating RPT with SSTR2 agonists [^177^Lu]Lu-DOTATATE and [^225^Ac]Ac-DOTATATE in patients with SCLC are ongoing (NCT05142696 and NCT05595460). These studies may provide additional information on the tolerability, side effects, and efficacy of RPT in patients with SCLC.

## CONCLUSION

This study demonstrates that [^68^Ga]Ga-SSO120 PET/CT can detect SCLC and LCNEC during and after chemotherapy. These findings support further therapeutic studies of [^177^Lu]Lu-SSO110 RPT in patients with SCLC and LCNEC, either after first-line chemotherapy or at later stages, such as at the time of relapse.

## DISCLOSURE

This work was financially supported by Ariceum-Therapeutics GmbH. Ariceum-Therapeutics GmbH had no influence on this study or its publication. Gitte Persson reports a research grant from Varian Medical Systems outside the submitted work. The graphical abstract was created in Biorender. No other potential conflict of interest relevant to this paper was reported.

## Notes

KEY POINTS **QUESTION:** Can [^68^Ga]Ga-SSO120 PET/CT detect SCLC and LCNEC? **PERTINENT FINDINGS:** In this prospective clinical trial, [^68^Ga]Ga-SSO120 PET/CT successfully visualized SCLC and LCNEC lesions during and after chemotherapy, detecting malignant tumors in 18 of 19 patients. **IMPLICATIONS FOR PATIENT CARE:** Therapeutic studies with [^177^Lu]Lu-SSO110, the theranostic companion of [^68^Ga]Ga-SSO120, PET are warranted.

## References

[1] Danish Lung Cancer Registry annual report 2022 [in Danish]. Danish Healthcare Quality Institute website. https://www.sundk.dk/media/54lhcg4f/9cab2e132e434b56896364f4538a1104.pdf. Published June 23, 2023. Accessed December 22, 2025.

[2] Xu F Chen K Lu C. Large Cell neuroendocrine carcinoma shares similarity with small cell carcinoma on the basis of clinical and pathological features. Transl Oncol. 2019;12:646–655. 30818166 10.1016/j.tranon.2019.01.004 PMC6393706

[3] Dingemans AC Fruh M Ardizzoni A.; ESMO Guidelines Committee. Small-cell lung cancer: ESMO clinical practice guidelines for diagnosis, treatment and follow-up. Ann Oncol. 2021;32:839–853. 33864941 10.1016/j.annonc.2021.03.207 PMC9464246

[4] Andrini E Marchese PV De Biase D. Large cell neuroendocrine carcinoma of the lung: current understanding and challenges. J Clin Med. 2022;11:1461. 35268551 10.3390/jcm11051461 PMC8911276

[5] Rindi G Klimstra DS Abedi-Ardekani B. A common classification framework for neuroendocrine neoplasms: an International Agency for Research on Cancer (IARC) and World Health Organization (WHO) expert consensus proposal. Mod Pathol. 2018;31:1770–1786. 30140036 10.1038/s41379-018-0110-y PMC6265262

[6] Dam G Grønbæk H Sundlöv A. Nordic 2023 guidelines for the diagnosis and treatment of lung neuroendocrine neoplasms. Acta Oncol. 2023;62:431–437. 37194281 10.1080/0284186X.2023.2212411

[7] Lehman JM Hoeksema MD Staub J. Somatostatin receptor 2 signaling promotes growth and tumor survival in small-cell lung cancer. Int J Cancer. 2019;144:1104–1114. 30152518 10.1002/ijc.31771 PMC6448409

[8] Bozkurt MF Virgolini I Balogova S. Guideline for PET/CT imaging of neuroendocrine neoplasms with ^68^Ga-DOTA-conjugated somatostatin receptor targeting peptides and ^18^F-DOPA. Eur J Nucl Med Mol Imaging. 2017;44:1588–1601. 28547177 10.1007/s00259-017-3728-y

[9] Lapa C Hanscheid H Wild V. Somatostatin receptor expression in small cell lung cancer as a prognostic marker and a target for peptide receptor radionuclide therapy. Oncotarget. 2016;7:20033–20040. 26936994 10.18632/oncotarget.7706 PMC4991436

[10] Nicolas GP Schreiter N Kaul F. Sensitivity comparison of ^68^Ga-OPS202 and ^68^Ga-DOTATOC PET/CT in patients with gastroenteropancreatic neuroendocrine tumors: a prospective phase II imaging study. J Nucl Med. 2018;59:915–921. 29191855 10.2967/jnumed.117.199760

[11] Wild D Fani M Fischer R. Comparison of somatostatin receptor agonist and antagonist for peptide receptor radionuclide therapy: a pilot study. J Nucl Med. 2014;55:1248–1252. 24963127 10.2967/jnumed.114.138834

[12] Fani M Nicolas GP Wild D. Somatostatin receptor antagonists for imaging and therapy. J Nucl Med. 2017;58(suppl 2):61S–66S. 28864614 10.2967/jnumed.116.186783

[13] Nicolas GP Beykan S Bouterfa H. Safety, biodistribution, and radiation dosimetry of ^68^Ga-OPS202 in patients with gastroenteropancreatic neuroendocrine tumors: a prospective phase I imaging study. J Nucl Med. 2018;59:909–914. 29025985 10.2967/jnumed.117.199737

[14] Kersting D Sandach P Sraieb M. ^68^Ga-SSO-120 PET for initial staging of small cell lung cancer patients: a single-center retrospective study. J Nucl Med. 2023;64:1540–1549. 37474272 10.2967/jnumed.123.265664

[15] Keller SH Jakoby B Svalling S Kjaer A Højgaard L Klausen TL. Cross-calibration of the Siemens mMR: easily acquired accurate PET phantom measurements, long-term stability and reproducibility. EJNMMI Phys. 2016;3:11. 27387738 10.1186/s40658-016-0146-3 PMC4936986

[16] Barrington SF Zwezerijnen B de Vet HCW. Automated segmentation of baseline metabolic total tumor burden in diffuse large B-cell lymphoma: which method is most successful? a study on behalf of the PETRA Consortium. J Nucl Med. 2021;62:332–337. 32680929 10.2967/jnumed.119.238923 PMC8049348

[17] Tricarico P Chardin D Martin N. Total metabolic tumor volume on ^18^F-FDG PET/CT is a game-changer for patients with metastatic lung cancer treated with immunotherapy. J Immunother Cancer. 2024;12:e007628. 38649279 10.1136/jitc-2023-007628 PMC11043703

[18] Mavroeidi IA Romanowicz A Haake T. Theranostics with somatostatin receptor antagonists in SCLC: correlation of ^68^Ga-SSO120 PET with immunohistochemistry and survival. Theranostics. 2024;14:5400–5412. 39310095 10.7150/thno.98819 PMC11413793

[19] Virgolini I Bahri S Kjaer A. A randomized, factorial phase II study to determine the optimal dosing regimen for (68)Ga-satoreotide trizoxetan as an imaging agent in patients with gastroenteropancreatic neuroendocrine tumors. J Nucl Med. 2022;63:376–383. 34215673 10.2967/jnumed.121.261936 PMC8978200

[20] Lin Z Zhu W Zhang J Miao W Yao S Huo L. Head-to-head comparison of ^68^Ga-NODAGA-JR11 and ^68^Ga-DOTATATE PET/CT in patients with metastatic, well-differentiated neuroendocrine tumors: interim analysis of a prospective bicenter study. J Nucl Med. 2023;64:1406–1411. 37474267 10.2967/jnumed.122.264890

[21] Wild D Grønbæk H Navalkissoor S. A phase I/II study of the safety and efficacy of [^177^Lu]Lu-satoreotide tetraxetan in advanced somatostatin receptor-positive neuroendocrine tumours. Eur J Nucl Med Mol Imaging. 2023;51:183–195. 37721581 10.1007/s00259-023-06383-1 PMC10684626

[22] Schurrle SB Eberlein U Ansquer C. Dosimetry and pharmacokinetics of [^177^Lu]Lu-satoreotide tetraxetan in patients with progressive neuroendocrine tumours. Eur J Nucl Med Mol Imaging. 2024;51:2428–2441. 38528164 10.1007/s00259-024-06682-1 PMC11178655

[23] Werner RA Solnes LB Javadi MS. SSTR-RADS version 1.0 as a reporting system for SSTR PET imaging and selection of potential PRRT candidates: a proposed standardization framework. J Nucl Med. 2018;59:1085–1091. 29572257 10.2967/jnumed.117.206631

[24] Krebs S O’Donoghue JA Biegel E. Comparison of ^68^Ga-DOTA-JR11 PET/CT with dosimetric ^177^Lu-satoreotide tetraxetan (^177^Lu-DOTA-JR11) SPECT/CT in patients with metastatic neuroendocrine tumors undergoing peptide receptor radionuclide therapy. Eur J Nucl Med Mol Imaging. 2020;47:3047–3057. 32378020 10.1007/s00259-020-04832-9 PMC7644587

